# Acousto-optic modulation of photonic bound state in the continuum

**DOI:** 10.1038/s41377-019-0231-1

**Published:** 2020-01-01

**Authors:** Zejie Yu, Xiankai Sun

**Affiliations:** Department of Electronic Engineering, The Chinese University of Hong Kong, Shatin, New Territories, Hong Kong SAR, China

**Keywords:** Photonic devices, Integrated optics

## Abstract

Photonic bound states in the continuum (BICs) have recently been studied in various systems and have found wide applications in sensors, lasers, and filters. Applying BICs in photonic integrated circuits enables low-loss light guidance and routing in low-refractive-index waveguides on high-refractive-index substrates, which opens a new avenue for integrated photonics with functional single-crystal materials. Here, we demonstrate high-quality integrated lithium niobate microcavities inside which the photonic BIC modes circulate and further modulate these BIC modes acousto-optically by using piezoelectrically actuated surface acoustic waves at microwave frequencies. With a high acousto-optic modulation frequency, the acousto-optic coupling is well situated in the resolved-sideband regime. This leads to coherent coupling between microwave and optical photons, which is exhibited by the observed electro-acousto-optically induced transparency and absorption. Therefore, our devices serve as a paradigm for manipulating and controlling photonic BICs on a chip, which will enable many other applications of photonic BICs in the areas of microwave photonics and quantum information processing.

## Tuning trapped light for integrated photonics

Light waves trapped within an integrated circuit could pave the way for microwave photonics and quantum information processing applications. Zejie Yu and Xiankai Sun of The Chinese University of Hong Kong demonstrated controlled interactions between sound waves and locally trapped light waves that could be used in integrated photonic circuits to reduce the amount of light loss during signal transmission. The researchers fabricated a device made of a low-refractive index, polymer waveguide placed on top of a lithium niobate film. Microwaves excited surface sound waves that changed the film’s refractive index and modulated the properties of locally trapped light moving inside the waveguide. Tuning the laser that introduces light into the device ultimately causes the lithium niobate film to switch between transparency and light absorbency.

## Introduction

The concept of “bound states in the continuum” (BICs) was first proposed by von Neumann and Wigner^1^ in 1929 with mathematical construction of a three-dimensional potential that can support perfectly confined states in a continuous band. The radiation loss of these confined states can be eliminated by destructive inference with the continuous modes. These phenomena have been observed in many physical systems^2^, including acoustics^3–7^, electronics^8–13^, and photonics^14–26^. Recently, the advancement of nanofabrication technologies has triggered the rapid development of BICs in photonics, enabling real applications in the areas of sensors^27,28^, lasers^15^, and filters^29^. Additionally, harnessing BICs in photonic integrated circuits (PICs) allows for low-loss light guidance and routing with a low-refractive-index waveguide on a high-refractive-index substrate. The light guided by the low-refractive-index waveguide can be confined to a region of the high-refractive-index substrate below the low-refractive-index waveguide^17^. PICs operating under the BIC principle do not require patterning micro- or nanostructures in the functional photonic material. Without the stringent requirement of high-quality etching, many single-crystal materials that exhibit excellent optical functionalities in bulk form can now be introduced to the integrated photonic platform.

Acousto-optics, such as Brillouin scattering, involves the study of phonon–photon interactions based on changes in the refractive index of a medium due to the presence of acoustic waves in that medium. It has found wide applications in various areas, such as nonreciprocal light transmission^30^, modulation^31^, frequency shifting^32,33^, and signal processing^34,35^. Surface acoustic waves (SAWs) that propagate on surfaces of a thin-film piezoelectric material can be confined within a thickness less than the acoustic wavelength, producing phonons with a very high density in the region near the surface. The small acoustic modal area, which is comparable to the optical modal area, results in a large overlap between the two modes in photonic waveguides. Therefore, SAWs can be used to achieve strong acousto–optic interactions in nanophotonic devices^36–39^. In addition, SAWs can be excited electromechanically, with the acoustic frequency reaching tens of GHz in piezoelectric materials^36^.

Lithium niobate (LiNbO_3_) has large piezoelectric coefficients and is optically transparent over a wide wavelength range. It can be used to generate SAWs efficiently and support photonic cavities with high quality factors. Therefore, LiNbO_3_ is an ideal platform for research on phonon–photon interactions. As PICs operating under the BIC mechanism allow for flexible selection of piezoelectric materials, LiNbO_3_ can be adopted to fabricate high-quality photonic microcavities on a chip without the need for etching. On the other hand, SAWs can be efficiently excited and propagate smoothly in the unetched LiNbO_3_ thin film without suffering from the reflection or scattering losses that would inevitably be introduced by etched structures. Therefore, harnessing BICs in PICs on a LiNbO_3_-on-insulator platform enables strong interactions of optical and acoustic waves to achieve the dynamic control of photonic BICs.

In this work, we demonstrated a high-quality photonic microcavity based on the BIC mechanism, which is integrated with an SAW interdigital transducer (IDT) monolithically on a thin-film LiNbO_3_-on-insulator platform. The BIC mechanism enables a cavity with an intrinsic optical quality factor higher than 500,000, which is constructed simply by patterning low-refractive-index waveguides on the high-refractive-index LiNbO_3_ substrate without facing the challenge of high-quality etching of LiNbO_3_. Meanwhile, SAWs can be efficiently excited and propagate smoothly in the unetched LiNbO_3_ thin film. We demonstrated for the first time acousto-optic modulation of the photonic BIC mode with modulation frequencies beyond 4 GHz. The combination of the high frequency of the SAW and the sub-GHz linewidth of the cavity resonance enables acousto-optic coupling in the resolved-sideband regime. As a result, we obtained coherent coupling between microwave and optical photons, as evidenced by the observed electro-acousto-optically induced transparency and absorption.

## Results

**Fig. 1:**
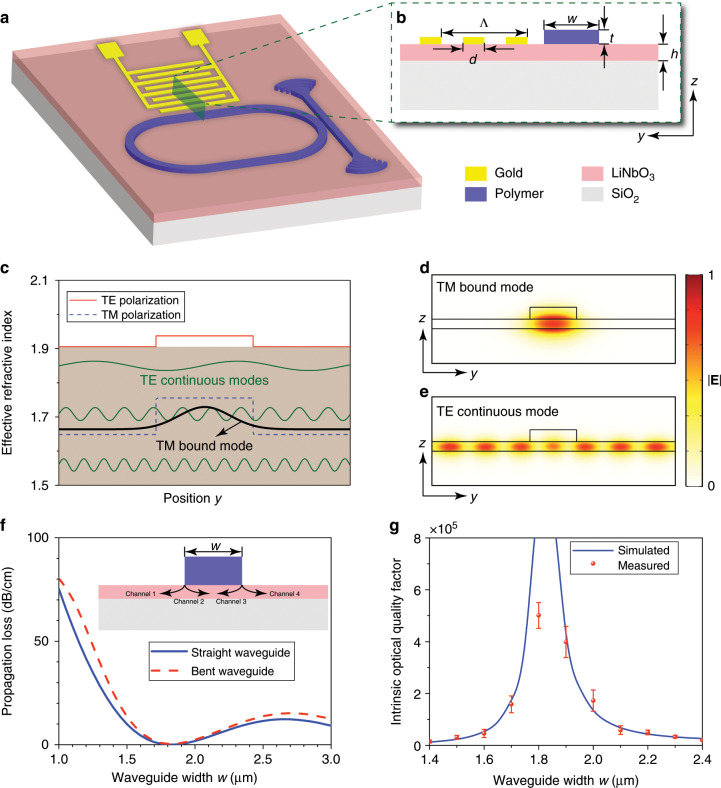
Design and experimental verification of photonic BIC. **a** Schematic of the entire device. The photonic microcavity is constructed from a low-refractive-index racetrack-shaped polymer waveguide (blue) on a high-refractive-index LiNbO_3_ (pink) substrate. The SAW interdigital transducer made of Au electrodes (yellow) is placed near a straight section of the racetrack microcavity. **b** Cross section of the waveguide supporting the photonic BIC mode. Λ and *d* are the period and width of the SAW interdigital transducer’s fingers, respectively, *w* and *t* are the width and thickness of the low-refractive-index polymer waveguide, respectively, and *h* is the thickness of the LiNbO_3_ film. **c** Effective refractive index distribution of the fundamental TE and TM modes in the waveguide in **b**. **d**, **e** Electric field |**E**| profiles of the TM bound mode (**d**) and a TE continuous mode (**e**) supported by the waveguide in **b**. **f** Simulated propagation loss of the TM bound mode in the straight (solid) and bent (dashed) section of the racetrack microcavity as a function of the waveguide width *w*. **g** Simulated (blue line) and measured (red dots) intrinsic optical quality factor of the cavity resonance as a function of the waveguide width *w*.

Figure 1a illustrates the device structure of an SAW IDT monolithically integrated with a photonic microcavity that supports BIC modes on a LiNbO_3_ platform, where the yellow part denotes the SAW IDT made of gold (Au), the blue part denotes the low-refractive-index waveguide and cavity made of electron-beam resist ZEP520A, the pink part denotes the high-refractive-index LiNbO_3_ substrate, and the grey part denotes SiO_2_. Figure 1b shows the cross section of the waveguide with a low-refractive-index material on a high-refractive-index substrate. According to conventional wisdom, low-refractive-index waveguides on high-refractive-index substrates cannot support propagating light modes without any optical loss because of inevitable dissipation to the substrate continuum. This phenomenon can be explained by the effective refractive index distributions for the TE (red solid) and TM (blue dashed) polarisations at a wavelength of 1.55 μm, as shown in Fig. 1c. The strong birefringence induced by the thin slab [TE (TM) effective refractive index is ~1.9 (~1.65)] causes the effective refractive index for the TM polarisation to lie below that for the TE polarisation. By making an analogy between the Schrödinger equation and the Helmholtz equation, one can find that the TM bound mode lies in the TE continuous spectrum. Therefore, numerous TE continuous modes (green lines in Fig. 1c) coexist with the TM bound mode (black line in Fig. 1c) in the LiNbO_3_ thin film, and the inevitable coupling between them results in optical loss to the TM bound mode (see Sec. 1 of the Supplementary Information). Figure 1d, e show the modal profiles (|**E**|) of the TM bound and TE continuous modes, respectively.

Defying conventional wisdom, the BIC mechanism predicts zero propagation loss in a waveguide structure, as shown in Fig. 1b ^17^. In this waveguide, the loss of the TM bound mode to the TE continuum occurs at the two waveguide edges, as illustrated in the inset of Fig. 1f. The loss at each edge originates from the coupling of the TM bound mode with the left-going (Channels 1 and 3) and right-going (Channels 2 and 4) TE continuous modes. If the losses via Channels 1 (2) and 3 (4) interfere destructively and cancel each other out, then the total loss of the TM bound mode to the TE continuum can be reduced to zero, leading to a lossless TM bound mode, which is the desired BIC. The inference of losses via Channels 1 (2) and 3 (4) depends on the phase difference caused by the finite width of the waveguide; thus, the BIC can be obtained just by optimising the waveguide width *w*. For a straight waveguide, the propagation length of the TM bound mode as a function of the waveguide width can be expressed as *L* ∝ *w*^2^/sin^2^(*k*_*y*_*w*/2), with *k*_*y*_ being the *y* component of the wave vector of the TE continuous mode, which matches that of the TM bound mode^17^. For a bent waveguide, the optical loss of the TM bound mode to the TE continuum of the high-refractive-index slab can be estimated from |*J*_*q*_(*nk*_0_*R*) – *ξJ*_*q*_(*nk*_0_(*R* – *w*))|, where *J*_*q*_ is the *q*th Bessel function, with *q* being the azimuthal mode number, and *ξ* is the ratio of the electric field intensities at the two edges of the waveguide^17^. Here, the optical loss depends not only on the waveguide width *w* but also on the bend radius *R*. It should be noted that although the loss to the substrate continuum can be eliminated by using the principle of BIC, the other loss channels, such as bending leakage to the free-space continuum, still cannot be avoided. Although the bending leakage cannot be reduced to absolute zero, it decreases exponentially with increasing bend radius and becomes negligibly small when the bend radius is sufficiently large. Therefore, when the BIC is obtained in practical experiments, the dissipation of photons will be limited only by the material absorption and fabrication imperfection rather than the radiation loss to the substrate. Since the waveguide width *w* necessary for a bent waveguide to achieve a BIC depends on the bend radius^17^, we chose the bend radius to be 200 μm, which is sufficiently large such that a BIC can be obtained simultaneously in both the straight and bent sections of a racetrack microcavity. Figure 1f plots the simulated propagation loss for light at a wavelength of 1.55 μm as a function of the waveguide width *w* in the straight (blue solid) and bent (red dashed) sections. The detailed simulation parameters are presented in Sec. 2 of the Supplementary Information. It is clear that the propagation loss can be reduced to zero, and thus, the desired BIC is obtained at the same waveguide width for both the straight and bent waveguides. A racetrack microcavity, as shown in Fig. 1a, is then constructed by connecting the straight and bent waveguides of the same waveguide width. Figure 1g plots the simulated (blue line) and measured (red dots) intrinsic optical quality factor of the racetrack microcavity as a function of the waveguide width *w*, where the measured values agree well with the simulated results. The simulated quality factors were evaluated from the propagation loss of the straight and bent waveguides, and the measured quality factors were obtained by fitting the experimental optical transmission spectra of the racetrack microcavities. It is clear that the optical quality factor depends crucially on the waveguide width *w*, and the highest value (corresponding to the desired BIC) is achieved at a certain waveguide width, which verifies the existence of the BIC in our structure.

**Fig. 2:**
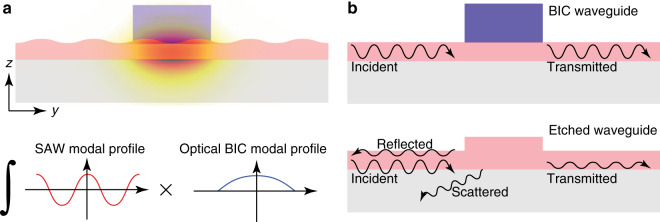
Acousto-optic coupling between the surface acoustic waves and photonic BIC modes that are circulating and resonating inside the photonic microcavity. **a** Illustration of the interaction between the SAW travelling along the *y* direction and the photonic BIC mode travelling along the *x* direction in the waveguide. The simulated modal field (|**E**|) of the BIC mode is superimposed onto the structure profile. A large overlap integral of the SAW mode and the photonic BIC mode yields strong acousto-optic coupling. **b** Illustration of an SAW propagating across a BIC waveguide (upper panel) and an etched waveguide (lower panel). The etched waveguide causes reflection and scattering of the incident SAW, but the BIC waveguide does not.

LiNbO_3_ is an ideal material for the efficient excitation of SAWs because of its strong piezoelectricity and high sound velocity. Meanwhile, the wavelength of ultrahigh-frequency SAWs can be as small as submicrometre to achieve a high modulation frequency in highly integrated acousto-optic devices. The scheme of acousto-optic modulation is illustrated in the upper panel of Fig. 2a. The propagating surface acoustic waves induce periodic strain fields inside the film, which transversely modulate the phase of the propagating optical mode in the waveguide by changing the refractive index of LiNbO_3_ through a combination of elasto-optic and electro-optic effects. Since |*E*_*y*_| is much smaller than |*E*_*z*_| for the TM bound mode, the overlap integral of the SAW strain field with the modal electric field of the optical waveguide can be expressed as^37^

$$\Gamma = \frac{{\int\!\int} \left[ p_{12}S_1\left(y,z\right) + p_{13}S_3\left(y,z\right) \right]\left| E_z\left( {y,z} \right) \right|^{2}dydz}{{\int\!\int} \left| E_z\left(y,z \right) \right|^{2}dydz }$$

where *p*_*ij*_ is the elasto-optic coefficient tensor and *S*_*i*_ is the strain field tensor. To obtain strong acousto–optic interactions, the overlap integral between the fundamental TM bound mode in the waveguide and the density variation associated with the surface acoustic wave shown in the lower panel of Fig. 2a should be maximised. Figure 2b illustrates an SAW propagating across a BIC waveguide (upper panel) and an etched waveguide (lower panel). When an SAW impinges on the sidewalls of a conventional etched waveguide (lower panel), it will be reflected and scattered due to the discontinuity in the film thickness. By contrast, an SAW will propagate across a BIC waveguide (upper panel) smoothly because the polymer waveguide atop with very different acoustic properties from those of LiNbO_3_ has a negligible effect on the SAW propagating inside the LiNbO_3_ thin film. In addition, a uniform thickness across the entire LiNbO_3_ device layer results in a constant wavelength of SAWs during propagation, which facilitates control of the SAW modal profiles, as the SAW wavelength matches the period of the SAW IDT.

**Fig. 3:**
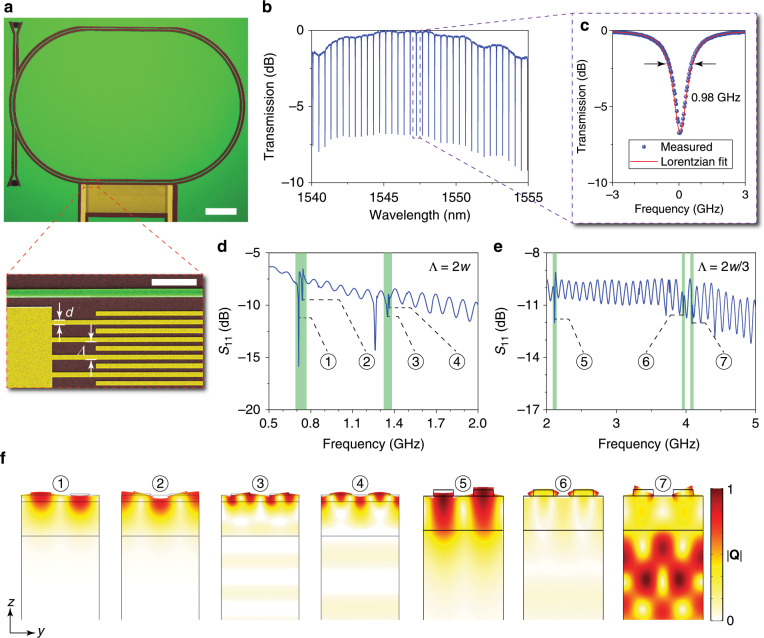
Characterisation of the photonic microcavity and SAW interdigital transducer. **a** Optical microscope and SEM images of a fabricated device for acousto-optic modulation, which consists of a racetrack photonic microcavity supporting circulating BIC modes and an interdigital transducer for exciting the SAW in the LiNbO_3_ thin film. Below the overview is a close-up of the SAW transducer showing the electrode fingers with a period Λ of 3.9 μm and finger width *d* of 925 nm. The scale bars in the overview and the close-up represent 50 and 10 μm, respectively. **b** Normalised optical transmission spectrum of the racetrack photonic microcavity measured from the nearby coupling waveguide. **c** Close-up spectrum of a cavity resonance (blue dots) fitted with a Lorentzian line shape (red line), indicating that the linewidth of the resonant mode is below 1 GHz. **d**, **e** Measured *S*_11_ spectra of SAW interdigital transducers with Λ = 2*w* (**d**) and Λ = 2*w*/3 (**e**). The excited surface acoustic modes appear as dips in the spectra. **f** Cross-sectional displacement field |**Q**| profiles of the corresponding resonant surface acoustic modes at the dips in **d** and **e**.

We fabricated the devices on a 400-nm LiNbO_3_-on-insulator wafer with silicon as the substrate handle. Figure 3a shows an optical microscope image of the fabricated device. The width *w* of the waveguide constructing the racetrack photonic microcavity is 1.95 μm, and the width of the coupling waveguide is 2.1 μm. The effective refractive index of the TM bound mode is 1.73, and the cross-sectional effective area of the TM bound mode is ~1.74 μm^2^. We fabricated two types of SAW IDTs with periods Λ = 2*w* and 2*w*/3. The scanning electron microscope (SEM) image below the device overview in Fig. 3a shows the SAW IDT with Λ = 2*w*. The finger width of the SAW IDT is 925 nm. The SAW IDT excites surface acoustic waves propagating in the direction transverse to the straight section of the racetrack microcavity (Fig. 3a), which has the same length as the aperture of the SAW IDT to achieve maximal acousto–optic interaction. In addition, the two straight sections of the racetrack microcavity are separated by the polymer, which introduces a large propagation loss to the SAWs; thus, the SAWs generated from one side of the microcavity cannot arrive at the other side. The circulating photonic mode can be modulated within only one straight section of the microcavity rather than both straight sections, thus eliminating the possibility of a cancelled modulation effect due to a *π* phase shift between the two straight sections. The photonic properties of the device were characterised by coupling light into and out of the device via a pair of grating couplers because the grating couplers not only facilitate power coupling between the fibre and the chip, but also serve as polarisers enabling high-efficiency excitation of the fundamental TM bound mode in the on-chip waveguides^40^. The optical resonances of the racetrack microcavity can be observed in the normalised transmission spectrum (Fig. 3b) measured from the coupling waveguide. Figure 3c is a close-up of an optical resonance with a Lorentzian fit, which shows that the linewidth of the cavity resonance is below 1 GHz. We also characterised the SAW IDTs by reflection measurements in the microwave domain. The SAW IDT was in contact with a microwave coplanar probe, which was connected to a vector network analyser to record the *S*_11_ spectra. Figure 3d, e plot the *S*_11_ spectra measured from SAW IDTs with Λ = 2*w* and Λ = 2*w*/3, respectively. Acoustic modes with frequencies up to 4 GHz can be observed in these reflection spectra as prominent dips, indicated in the marked regions. Figure 3f plots the cross-sectional modal profiles of the corresponding resonant surface acoustic modes.

**Fig. 4:**
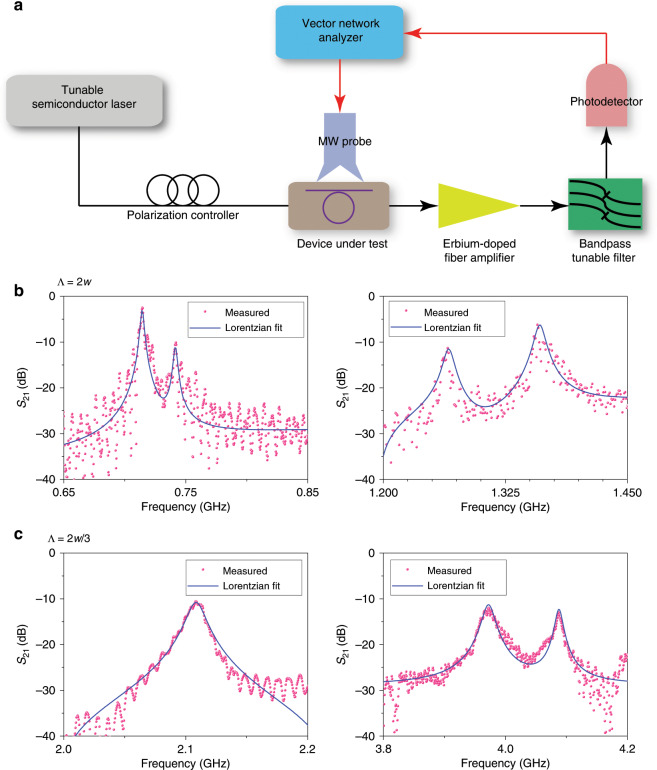
Acousto-optic modulation of the cavity resonant BIC modes. **a** Experimental set-up. **b**, **c** Measured *S*_21_ spectra for devices with SAW interdigital transducer finger period Λ = 2*w* (**b**) and Λ = 2*w*/3 (**c**). The red dots denote the recorded data points, and the blue lines are the corresponding Lorentzian fitting curves.

Figure 4a shows the experimental set-up used to measure the acousto–optic interaction of cavity-enhanced photonic BIC. Light from a tuneable semiconductor laser was sent over a single-mode fibre, with the polarisation state adjusted by a fibre polarisation controller, into the device under test via the input grating coupler. Meanwhile, a vector network analyser delivered a sinusoidal microwave signal to the SAW IDT through a microwave (MW) probe to excite the SAWs. The acousto-optically modulated light was coupled out of the device chip and then amplified by an erbium-doped fibre amplifier (EDFA), followed by a bandpass tuneable filter to filter out the excessive amplified spontaneous emission noise introduced by the EDFA. After that, the light signal was converted into the electrical domain by a high-speed photodetector and then sent back to the network analyser. With this configuration, the measured *S*_21_ transmission spectra show the frequency response of acousto-optic modulation. During measurement, the laser wavelength was slightly detuned from a cavity resonance to maximise the detected signal. Figure 4b, c plot the measured *S*_21_ spectra for devices with an SAW IDT of Λ = 2*w* and Λ = 2*w*/3, respectively. The red dots are the data recorded directly from the network analyser, and the blue lines are the corresponding Lorentzian fits. Compared with the *S*_11_ reflection spectra of SAW IDTs in Fig. 3d, e, the perfect frequency match of the reflection dips in the *S*_11_ spectra and the modulation peaks in the *S*_21_ spectra confirms that the measured peaks result from the SAW modulations. In addition, the modulation frequency of an SAW with Λ = 2*w*/3 can exceed 4 GHz. In the presence of a sub-GHz linewidth of the cavity resonance in Fig. 3c, this high modulation frequency enables phonon–photon coupling in the resolved-sideband regime, a prerequisite for the coherent coupling of microwave and optical photons and associated phenomena such as electro-acousto-optically induced transparency and absorption.

**Fig. 5:**
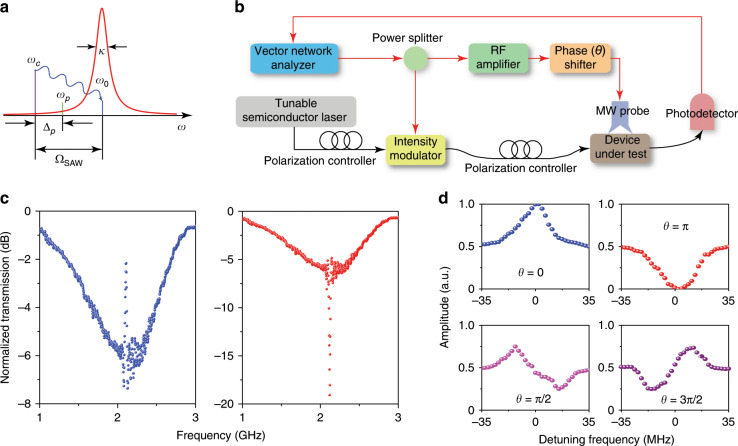
Coherent coupling between microwave and optical photons exhibited by electro-acousto-optically induced transparency and absorption. **a** Illustration of the three-wave mixing process of the control light (*ω*_*c*_), probe light (*ω*_*p*_), and SAW (Ω_SAW_). The cavity resonant frequency is *ω*_0_ with a decay rate of *κ*. **b** Homodyne measurement set-up. **c** Measured normalised transmission spectra of the probe light, showing the features of electro-acousto-optically induced transparency and absorption. When the SAW-scattered anti-Stokes component of the control light is in phase with the probe light, the constructive interference leads to a narrow transparency window with a bandwidth matching the linewidth of the surface acoustic mode (blue dots). When the SAW-scattered anti-Stokes component is *π* out of phase with the probe light, the destructive interference leads to enhanced cavity absorption (red dots). **d** Normalised transmission of the probe light when the phase shift *θ* is set at 0 (blue), *π*/2 (magenta), *π* (red), and 3*π*/2 (purple). When *θ* is at *π*/2 and 3*π*/2, the spectra take the shape of a Fano resonance.

The process of electro-acousto-optically induced transparency or absorption is illustrated in Fig. 5a, where the probe light (*ω*_*p*_) interferes with the Stokes or anti-Stokes sideband of the control light (*ω*_*c*_), which is generated by coherent interactions between the cavity photons and propagating phonons. The propagating phonons excited by the SAW IDT in our device can be confined to the LiNbO_3_ thin film with a high density. The constructive (destructive) interference between the probe light and the Stokes or anti-Stokes sideband of the control light results in a sharp transparency or absorption window in the transmission spectrum of the probe light. We investigated the coherent three-wave nonlinear process with the experimental set-up depicted in Fig. 5b. A laser was detuned from the cavity resonance at the SAW modal frequency (Ω_SAW_) to serve as the control light (*ω*_*c*_). Meanwhile, the intensity of the laser was also modulated with an electro-optic modulator to generate sidebands, producing a frequency tone at *ω*_*p*_ = *ω*_*c*_ + Δ_*p*_, which was used as the probe light. With this scheme, by varying the modulation frequency Δ_*p*_, one obtains the transmission spectrum of the cavity from the beating signal between the transmitted probe light (*ω*_*p*_) and the control light (*ω*_*c*_). When the modulation signal is also sent, after amplification and phase shifting, to the SAW IDT, an SAW of the same frequency is excited and propagates to the racetrack microcavity, yielding acousto-optic coupling among the three waves: the control light, the probe light, and the SAW. The phase of the SAW depends on the modulation signal sent to the SAW IDT, which is controlled accurately by a phase shifter. The interference between the probe light and SAW can be varied from constructive to destructive by tuning the phase shift *θ* of the modulation signal, resulting in electro-acousto-optically induced transparency or absorption (Fig. 5c). The transparency and absorption window width matches the SAW modulation bandwidth, as shown in Fig. 4c, because in this homodyne measurement scheme, the acoustic frequency and the probe detuning are synchronised. In addition to the demonstrated transparency and absorption, we also investigated acousto-optic coupling with different phase shifts. Figure 5d shows the results when the phase shift *θ* is set to 0, *π*/2, *π*, and 3*π*/2. It is clear that the interference can be tuned continuously from constructive to destructive and displays Fano-resonance-like line shapes in between.

## Discussion

In conclusion, acousto-optic modulation of cavity-enhanced photonic bound states in the continuum in both the unresolved- and resolved-sideband regimes has been demonstrated for the first time. Electro-acousto-optically induced transparency and absorption are also observed, indicating the strong coherent coupling between photons and phonons. The unique feature and main advantage of the present scheme are that by harnessing the low-loss light guidance under the BIC mechanism, the single-crystal LiNbO_3_ layer is free from etching, thus producing SAWs of uniform acoustic wavelengths and low acoustic propagation loss, which facilitates highly efficient phonon‒photon coupling. Therefore, our devices have demonstrated the advantages of a BIC-based integrated photonic platform for achieving phonon‒photon coupling on a chip. The strong phonon‒photon coupling obtained in this work can be harnessed to develop a wide range of Brillouin-scattering-based photonic applications, including delay lines, light storage, microwave signal processing, Brillouin lasers and amplifiers, and nonreciprocal light transmission. Additionally, the travelling acoustic waves in this work are electrically excited, being much stronger than those excited by optical methods. By using a piezoelectric material, it is not necessary to fabricate delicate suspended structures similar to those in conventional on-chip stimulated-Brillouin-scattering-based devices. Therefore, our demonstrated devices have great promise in achieving high performance in Brillouin-effect-based applications with a more robust architecture.

## Materials and methods

### Device fabrication

The devices were fabricated on a *z*-cut LiNbO_3_-on-insulator wafer purchased from NANOLN, where the nominal thickness of the LiNbO_3_ layer is 400 nm. We first fabricated the SAW IDTs with a lift-off process involving electron-beam lithography and gold deposition, where the thickness of the gold electrodes is 80 nm. Then, we performed a second step of electron-beam lithography to pattern the photonic waveguides, microcavities, and grating couplers in an electron-beam resist (ZEP520A), which serves as the polymer layer in Fig. 1a. The thickness of the electron-beam resist ZEP520A was controlled to be 500 nm by using a spinning speed of 2400 r/min during spin coating.

## Supplementary information

SUPPLEMENTARY INFORMATION for Acousto-optic modulation of photonic bound state in the continuum
