# Supplementary material for: Acousto-optic modulation of photonic bound state in the continuum
Source: Light Sci Appl. 2020 Jan 1;9:1. doi: 10.1038/s41377-019-0231-1 (PMC6946673; doi:10.1038/s41377-019-0231-1)
Supplement: Supplementary file 1 — SUPPLEMENTARY INFORMATION for Acousto-optic modulation of photonic bound state in the continuum [file 41377_2019_231_MOESM1_ESM.pdf]

# Supplementary Information for "Acousto-optic modulation of photonic bound state in the continuum"

Zejie Yu and Xiankai Sun*

*Department of Electronic Engineering, The Chinese University of Hong Kong, Shatin, New Territories, Hong Kong SAR, China*

*Corresponding author: xksun@cuhk.edu.hk*

## 1. Principle of light guidance in the BIC mode

Solving the Schrödinger equation for a quantum system with a finite potential well, one obtains the eigenstates with their energy eigenvalues lying inside the potential well. These eigenstates can be square integrable and are known as bound states of the quantum system. The other eigenstates with their energy eigenvalues outside the potential well are not square integrable. These eigenstates are known as continuous states, which are extended to infinity. Taking the analogy of the Schrödinger equation and the Helmholtz equation[S1,S2], the refractive index $n$ of a material determines the potential for photons inside that material as $-n^2k_0^2$, where $k_0$ is the wave number of light in the vacuum. Therefore, a higher (lower) effective refractive index leads to a lower (higher) potential for photons.

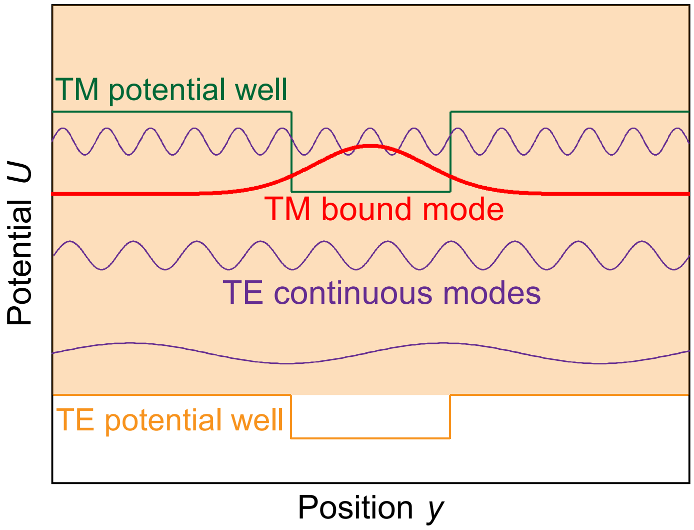


**Figure S1** | Photonic potential distribution of the hybrid waveguide structure shown in Fig. 1b. The yellow and green lines represent the potential wells for the TE and TM polarizations, respectively. The TM bound mode (red line) lies in the TE continuous spectrum.

Figure S1 plots the photonic potential distributions for the transverse electric (TE) and transverse magnetic (TM) polarizations in the hybrid waveguide structure (Fig. 1b), based on the effective refractive index distributions (Fig. 1c). The potential well for the TM polarization lies above that for the TE polarization. Therefore, the TM bound mode localized in the potential well for the TM polarization lies in the continuous spectrum of the TE polarization.

Usually, the TM bound mode is a leaky bound state due to its coupling with the TE continuous modes. It is well known that a perfect TM mode with purely vertically polarzied electric field ($E_z$) would not be coupled with the TE modes with purely horizontally polarized electric field ($E_y$), because of their zero modal overlap. However, the hybrid waveguide structure shown in Fig. 1b does not support a perfect TM bound mode. Figures S2a and S2b show the profiles of the vertical ($E_z$) and horizontal ($E_y$) components of the TM bound mode, respectively. The nonzero $E_y$ component in Fig. S2b indicates that the TM bound mode contains a slight portion of the TE polarization, which is induced by the broken symmetry of the rib waveguide. Therefore, the TM bound mode discussed in this paper is actually a quasi-TM mode instead of a perfect TM mode. Its nonzero $E_y$ component is responsible for its coupling with the TE continuous modes in the high-refractive-index substrate.

Defying this conventional wisdom, bound states in the continuum (BICs) refer to a type of eigenstates whose wavefunctions are square integrable yet the corresponding energy eigenvalues are above the potential well. Therefore, harnessing the BICs in the hybrid waveguide structure shown in Fig. 1b will enable lossless light guidance and propagation of the TM bound mode in the TE continuous spectrum.

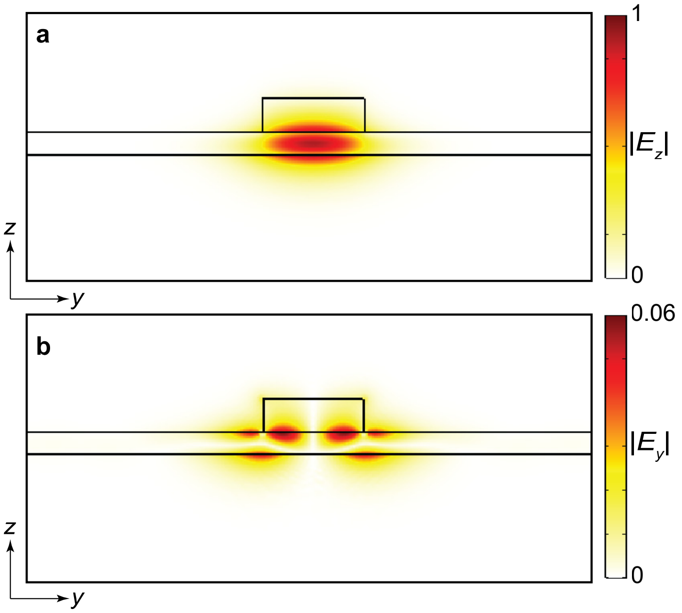


**Figure S2 |** Profiles of the $E_z$ (a) and $E_y$ (b) components of the TM bound mode.

## 2. Microcavities operating in the BIC mode

We calculated the propagation loss for the straight waveguide shown in Fig. S3a with a finite-element method in COMSOL[S3]. We set the refractive indices of $LiNbO_3$ $n_o = 2.21$ and $n_e = 2.13$ and the refractive index of polymer 1.54. For a 500-nm-thick low-refractive-index polymer waveguide on a 400-nm $LiNbO_3$-on-insulator substrate, the simulated propagation loss for light at the wavelength of 1.55 μm as a function of the waveguide width $w$ is shown in Fig. S3b. The propagation loss can reach zero for a certain combination of structural parameters. Figures S3c–S3f show the modal profiles of the TM bound mode at different waveguide widths. The radiation

loss to the substrate continuum can be reduced to zero as shown in Fig. S3e when the BIC condition is satisfied.

We also calculated the propagation loss for the bent waveguide shown in Fig. S4a with a finite-element method in COMSOL[S3]. Figure S4b shows the simulated propagation loss for light at the wavelength of 1.55 μm as a function of the bent waveguide width *w* when the waveguide bend radius *R* is 200 μm. The propagation loss can also reach zero for a certain combination of structural parameters. Figures S4c–S4f show the modal profiles of the TM bound mode at different waveguide widths. Similarly, the radiation loss to the substrate continuum can be reduced to zero as shown in Fig. S4e when the BIC condition is satisfied.

Although the experimental optical quality factors of our BIC-based microcavities are lower than those of devices fabricated by patterning and etching[S4], there is still much room for further improvement. The ultimate limit of our approach is determined by the residual material absorption of the substrate and the low-refractive-index waveguide. Presently, the quality factor of our devices is limited mainly by optical absorption from the residual solvent in the e-beam resist[S5]. Alternatively, we can adopt low-loss transparent dielectric materials such as $SiO_2$ or atomic-layer-deposited $Al_2O_3$ for the low-refractive-index waveguide on our BIC-based integration platform to improve the device performance. These dielectric materials are CMOS-compatible and have been adopted for producing optical cavities of ultrahigh quality factors. Our devices should benefit from significant improvement of optical quality factors when these materials are adopted.

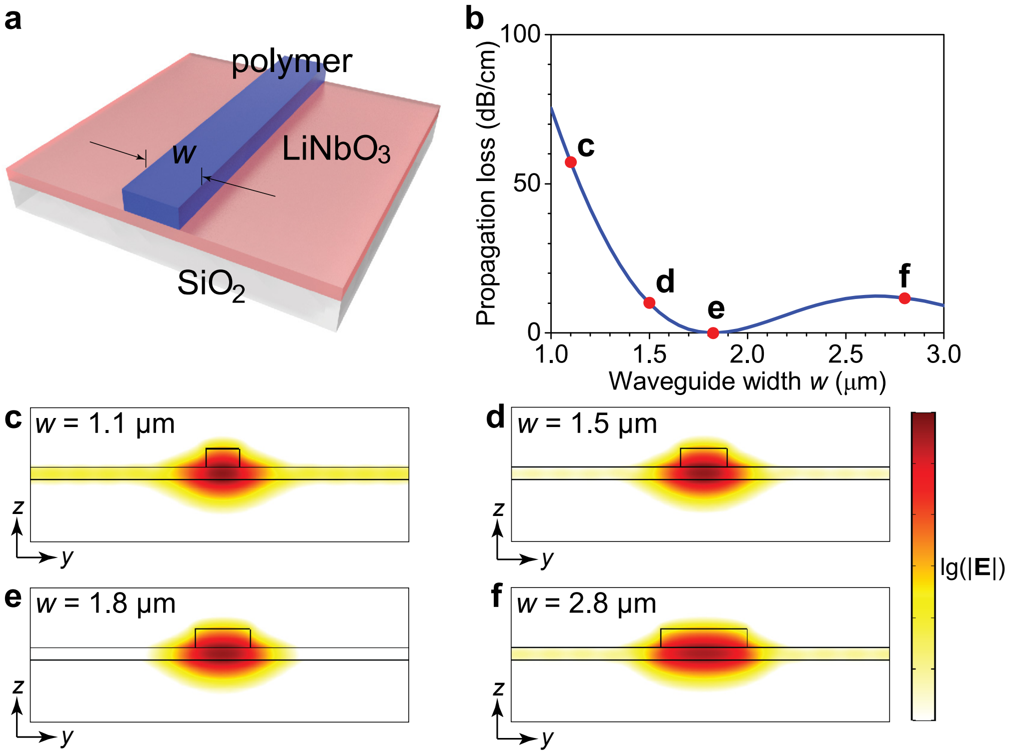


**Figure S3 |** (a) Illustration of a low-refractive-index straight waveguide on a high-refractive-index $LiNbO_3$ substrate. (b) Simulated propagation loss of the TM bound mode in the waveguide in (a) as a function of the waveguide width *w*. (c)–(f) Modal profiles of the TM bound mode at different waveguide widths.

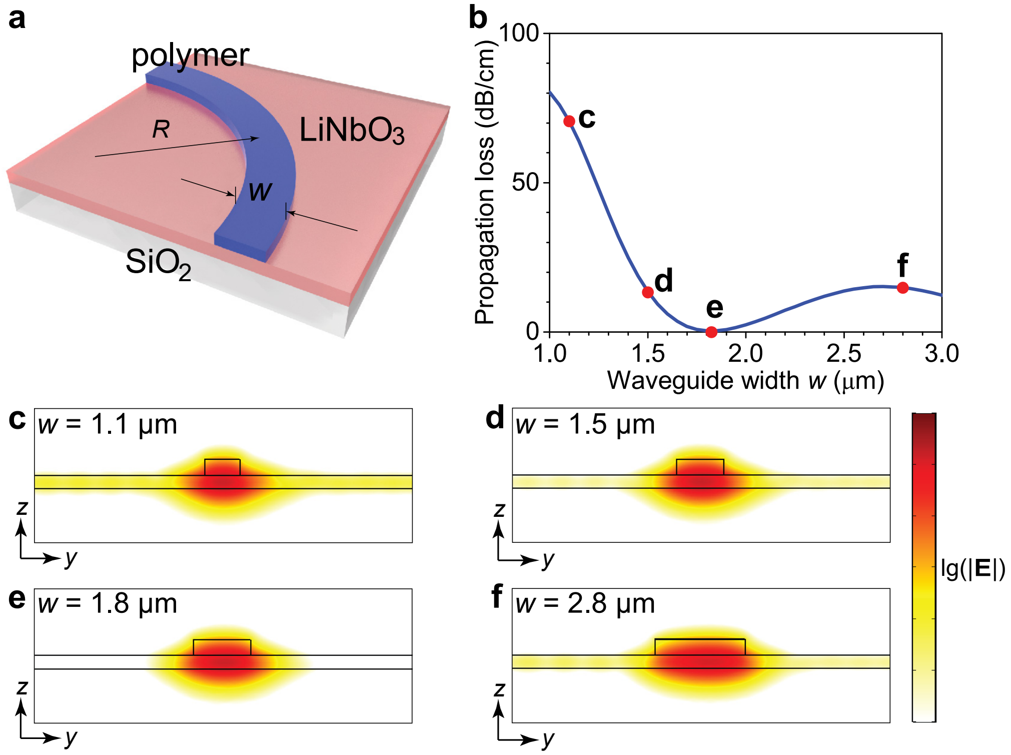


**Figure S4 |** (a) Illustration of a low-refractive-index bent waveguide on a high-refractive-index $LiNbO_3$ substrate. (b) Simulated propagation loss of the TM bound mode in the waveguide in (a) as a function of the waveguide width *w* when the waveguide bend radius *R* is 200 μm. (c)–(f) Modal profiles of the TM bound mode at different waveguide widths.

## References


S1. Longhi, S. Quantum-optical analogies using photonic structures. *Laser Photon. Rev.* **3**, 243–261 (2009).

S2. Dragoman, D. & Dragoman, M. *Quantum-Classical Analogies*. (Springer Science & Business Media, 2013).

S3. COMSOL Multiphysics® v. 4.3a. www.comsol.com. COMSOL AB, Stockholm, Sweden.

S4. Zhang, M., Wang, C., Cheng, R., Shams-Ansari, A. & Lončar, M. Monolithic ultra-high-*Q* lithium niobate microring resonator. *Optica* **4**, 1536–1537 (2017).

S5. Li, B.-B., Wang, Q.-Y., Xiao, Y.-F., Jiang, X.-F., Li, Y., Xiao, L. & Gong, Q. On chip, high-sensitivity thermal sensor based on high-*Q* polydimethylsiloxane-coated microresonator. *Appl. Phys. Lett.* **96**, 251109 (2010).
